# Supplementary material for: Amyloid-β Effects on Peripheral Nerve: A New Model System
Source: Int J Mol Sci. 2023 Sep 23;24(19):14488. doi: 10.3390/ijms241914488 (PMC10572603; doi:10.3390/ijms241914488)
Supplement: Supplementary file 1 [file ijms-24-14488-s001.zip › ijms-2629735-supplementary.pdf]

Supplementary Figures and Tables

Supplementary Figures

Supplementary Figure S1-Overall plan of the χ^2 analyses for the parameters extracted from the NAP waveform.

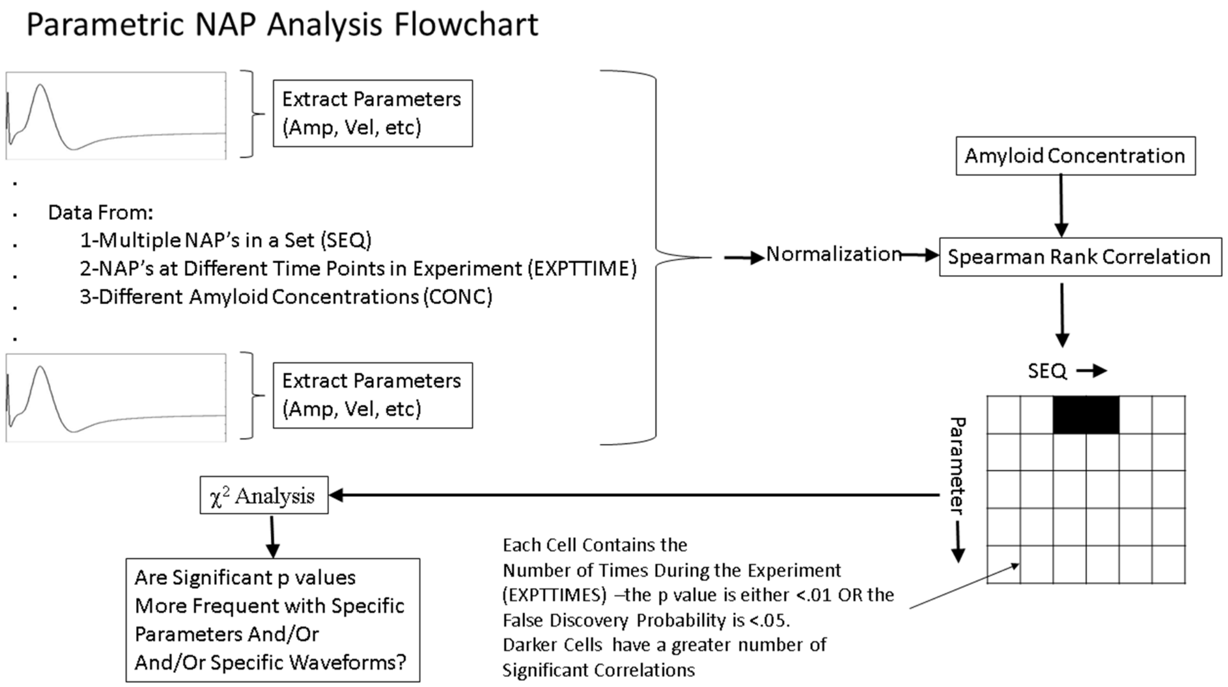

Supplementary Figure S2. Effect of Aβ42 on some of the parameters extracted from the NAP. There is a small tendency for duration to be slightly higher and decline to be larger for the intermediate concentrations but this is not statistically significant. Only the effect of concentration on amplitude of the NAP is significant.

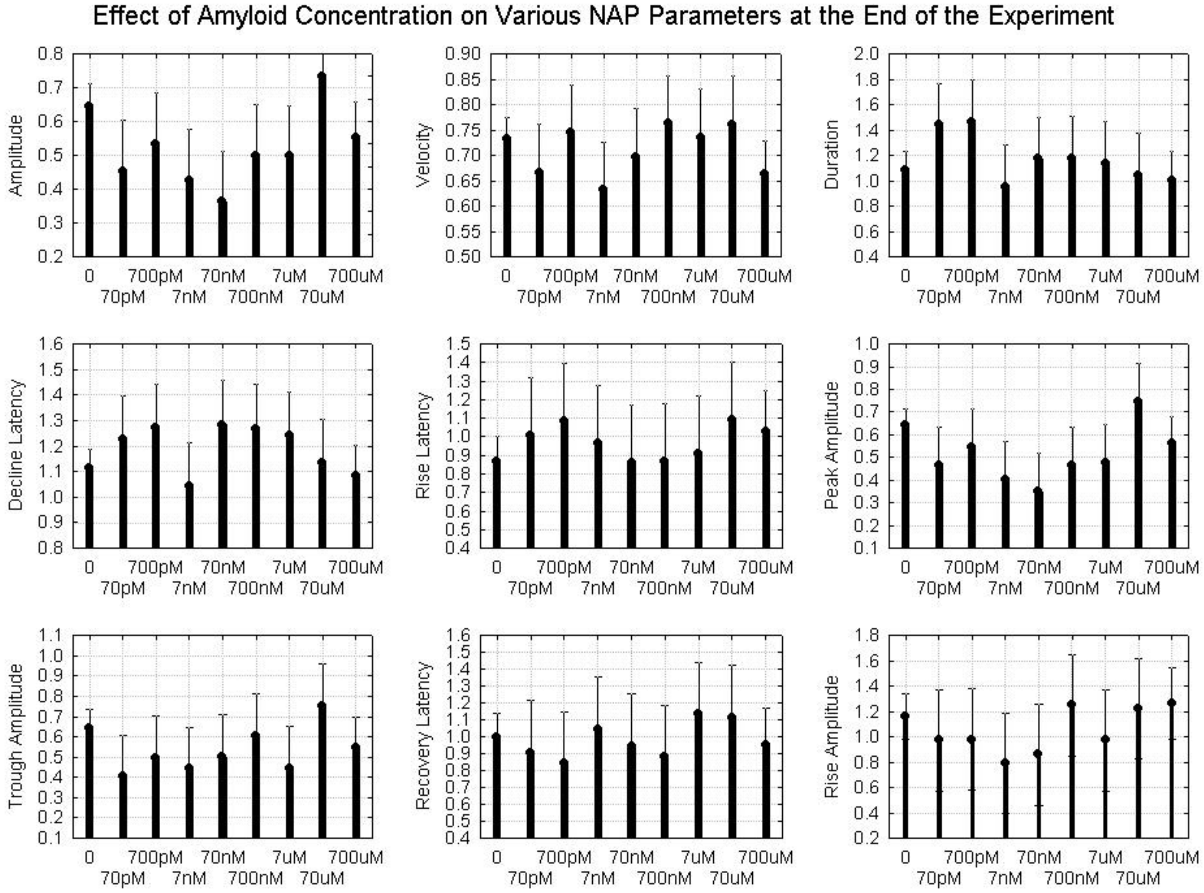

Supplementary Figure S3. The number of NAP parameters (out of 12x7) that showed a significant effect of Aβ42 concentration on the Spearman rank correlation testing as a function of the time during the experiment.

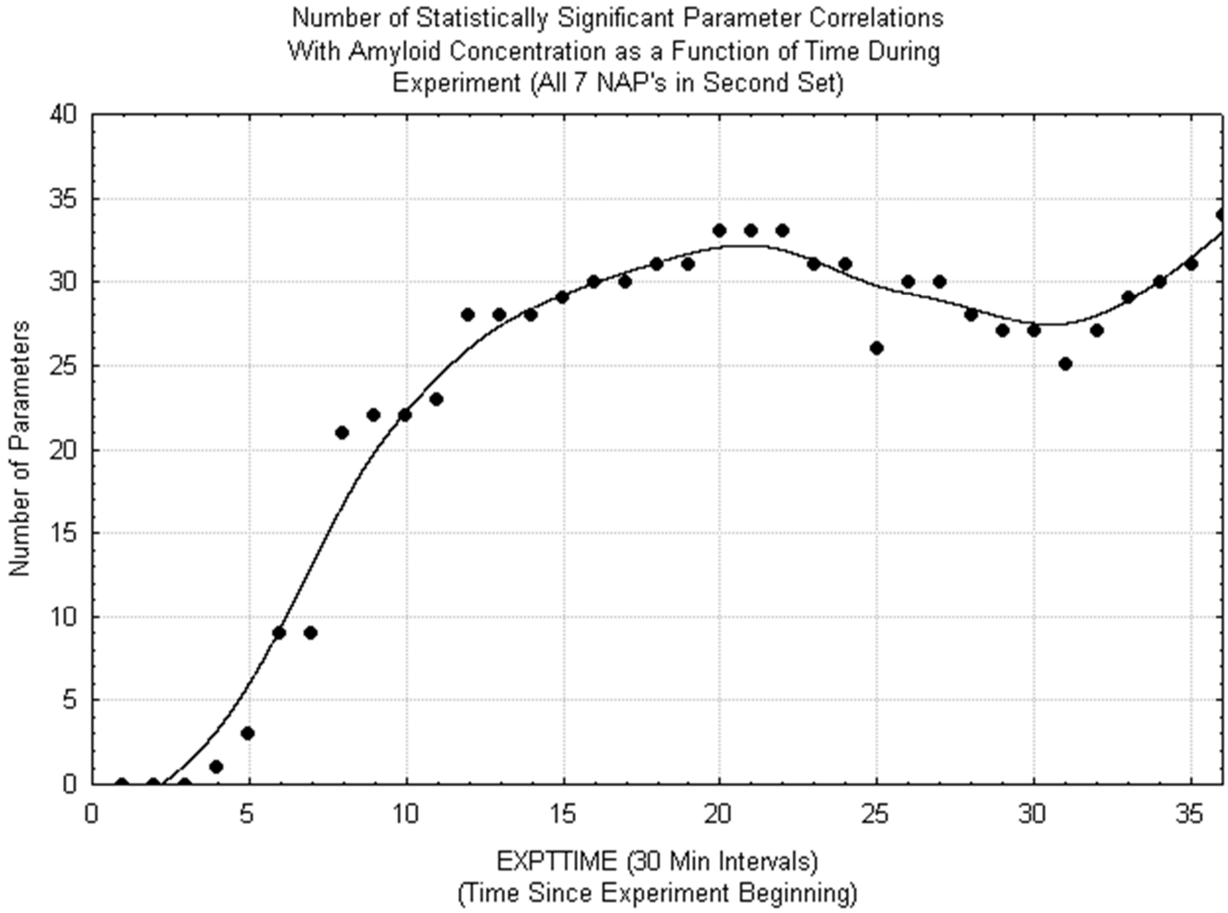

Supplementary Figure S4-. Normalized NAP waveforms averaged over all nerves in same Aβ42 concentration category at the end of the experiment for the 2mA and 15mA stimulus currents at 4msec ISI.

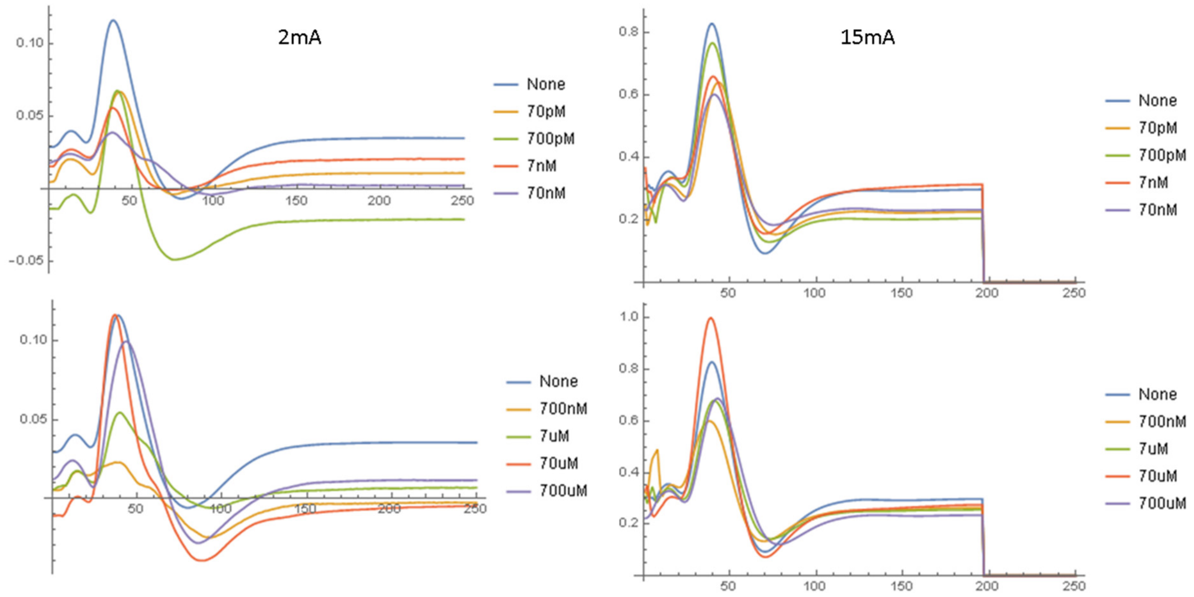

Supplementary Figure S5

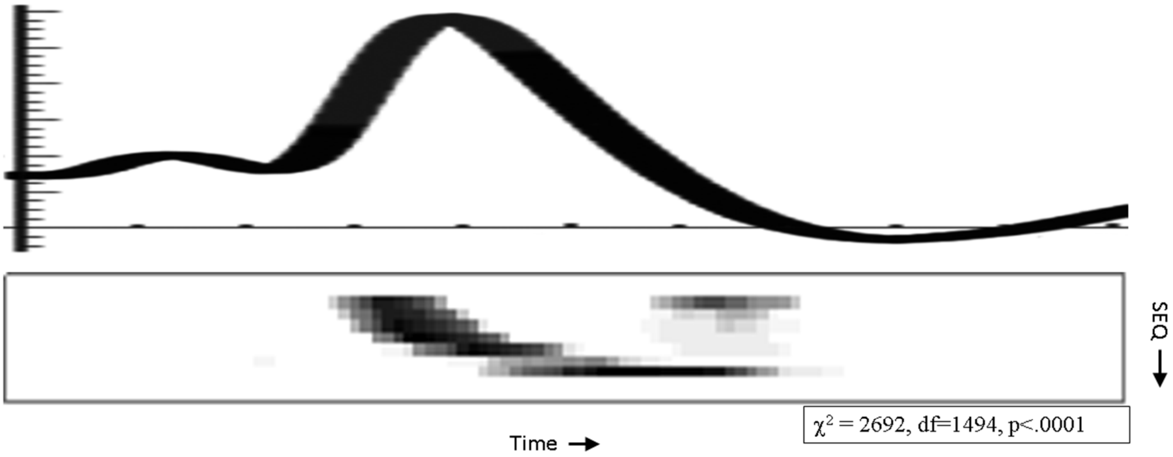

Table of the Number of Times during the Experiment (EXPTTIME) that the Rank Correlation Analysis Yields a significant p<.01 relationship between Amyloid concentration and the Voltage at that time Point for the given NAP waveform (SEQ) In the second stimulus set.

Supplemental Tables

Supplementary Table S1 - Simple ANOVA for each parameter

Analysis of the effects of Aβ42 on the parameters describing the first NAP in the second stimulus set as a function of Aβ42 concentration. The significance level for the main effects ANOVA is .05/12=.004 according to the Bonferroni correction. Confirmation of significant effects by non-parametric testing with the Kruskal-Wallis test is carried out as well. This suggests that most of the effects of Aβ42 are on the NAP amplitude.

Parameter	F(8,103)	p	Kruskal-Wallis
Amplitude	3.08	.004	.007
Velocity	1.2	.29	
Duration	1.3	.24	
Decline Latency	1.5	.18	
Rise Latency	.54	.82	
Peak Amplitude	2.8	.008	.005
Trough Amplitude	1.5	.16	
Recovery Latency	.49	.9	
Decline Amplitude	.96	.47	
Rise Amplitude	.82	.59	
Repolarization Amplitude	.68	.71	

Supplementary Table S2--Linear Regression Analysis

Data from the last time point in the experiment (EXPTTIME=36) for the first stimulus in the second set (15mA, 166ms ISI). Testing at p=.004 confirms that only amplitude and peak amplitude are significant. CONC is an ordinal variable 0-8 representing the concentration of Aβ42 (0,70pM,700pM,7nM,70nM,700nM,7μM,70μM,700μM).

	CONC			$CONC^2$			
Parameter	Slope (std)	t	p	Slope (std)	t	p	R^2
Amplitude	-.09 (.03)	3.5	.0006	.011 (.003)	3.35	.001	.1
Velocity	-.0015 (.016)	.09	.93	-.0002 (.002)	.11	.83	.005
Duration	.06 (.06)	1.1	.29	-.009 (.007)	1.4	.17	.02
Decline Latency	.06 (.03)	2.2	.03	-.008 (.004)	2.3	.02	.04
Rise Latency	.01 (.05)	.25	.81	.0004 (.007)	.07	.94	.01
Peak Amplitude	-.1 (.03)	3.5	.0006	.01 (.004)	3.4	.001	.1
Trough Amplitude	-.06 (.03)	1.8	.07	.008 (.005)	1.8	.08	.03
Recovery Latency	-.01 (.05)	.2	.84	.002 (.007)	.28	.77	.002
Decline Amplitude	-.12 (.07)	1.9	.06	.018 (.008)	2.12	.04	.04
Rise Amplitude	-.08 (.04)	2.2	.02	.011 (.005)	2.4	.02	.05
Repolarization Amplitude	-.079 (.05)	1.57	.12	.012 (.007)	1.84	.07	.04

Supplementary Table S3—Repeated measures ANOVA for the amplitude series stimuli. In this table the variable AMP is the amplitude of the stimulus and is the repeated measure with 6 levels (2mA, 3mA, 4mA, 6mA, 10mA, 15mA). The variable EXPTTIME is the time that the experiment has been running and CONC is the concentration of Aβ42 (0,70pm,700pm,7nm,70nm). This demonstrates significant interactions between the stimulus amplitude and the amyloid concentration (AMP*CONC) as well as significant effects of amyloid concentration on the effect of stimulus amplitude (CONC*AMP).

Factor	ndf1	ndf2	F	p
EXPTTIME	31	10720	51	<0.001
CONC	4	10720	122	<0.001
EXPTTIME*CONC	124	10720	0	1
AMP	5	10720	3054	<0.001
AMP*EXPTTIME	155	10720	18	<0.001
AMP*CONC	20	10720	14	<0.001
AMP*CONC*EXPTIME	620	10720	1	1

Supplementary Table S4—Repeated measures ANOVA for the ISI series stimuli. In this table the variable ISI is the interstimulus interval of the stimulus and is the repeated measure with 7 levels (166ms, 8ms, 4ms, 3ms, 2ms,1.5ms,1ms). The variable EXPTTIME is the time that the experiment has been running and CONC is the concentration of Aβ42 (0,70pm,700pm,7nm,70nm). This demonstrates significant interactions between the interstimulus interval and the amyloid concentration (ISI*CONC) as well as significant effects of amyloid concentration on the effect of ISI (CONC*ISI)

Factor	ndf1	ndf2	F	p
EXPTTIME	31	12864	52.2	<0.001
CONC	4	12864	121.7	<0.001
EXPTTIME*CONC	124	12864	0.50	1
ISI	5	12864	4625.69	<0.001
ISI*EXPTTIME	155	12864	19.76	<0.001
ISI*CONC	20	12864	25.8	<0.001
ISI*CONC*EXPTIME	620	12864	0.007	1

Consider a sequence of measurements $x_{i,j}(c); i=1...n, j=1...m$. In the specifics of this paper the $x_{ij}(c)$ represent the amplitude of the NAP at time interval i after the stimulus on trial j at an amyloid concentration of c. One way to determine the effect of c on x is to compute:

$$\bar{x}_i(c) = \frac{1}{m}\sum_{j=1}^{m} x_{ij}(c)$$

This leads to a set of curves:

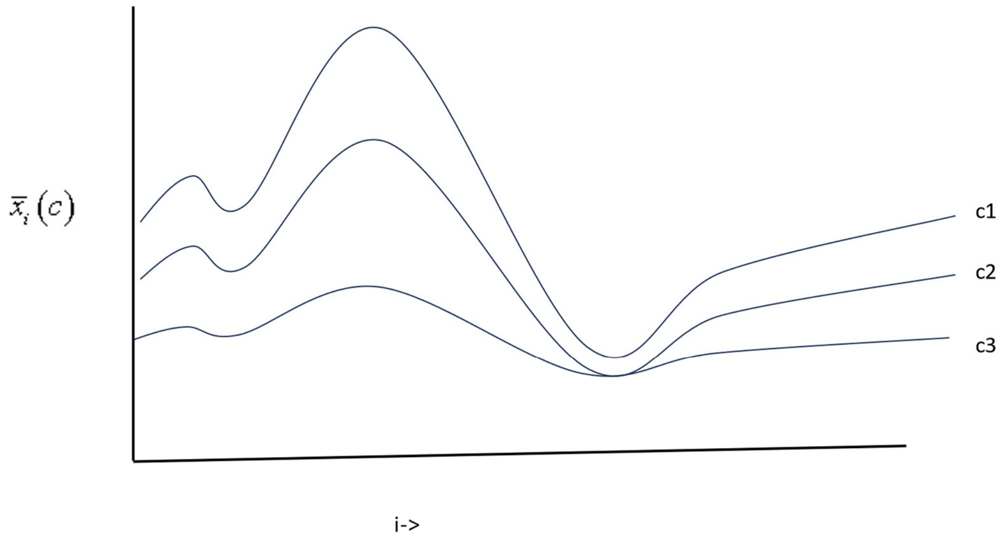

For three different values of c: c1,c2,c3. This is not in a form that is easily subjected to statistical analysis as the statistical variations have been removed in computation of the mean. One method of analyzing such curves for an effect of c is to extract a few features q $f_{kj}(c), k=1...q$ from each curve

$$f_{kj}(c) = g_k\left(\left\{x_{ij}(c); i=1...n\right\}\right)$$

Where g_k is the function that computes the value of the k'th feature from the each NAP recording. This may be an amplitude, latency or duration among other features. Since number of features is far less than the number of samples in the NAP q<<n, this allows us to carry out the statistical analysis with a reduced need to correct for multiple testing. It is reasonable to apply a statistical test to compare the difference in features at two concentrations to see if $f_k(c1), f_k(c2)$ are significantly different using the Bonferroni correction at testing at a p value of 0.05/q. For example, if the statistical test were a t-test then the test would be:

$$T_k(c1,c2)=\frac{\overline{f}_k(c1)-\overline{f}_k(c2)}{\sqrt{s_k(c1)^2+s_k(c2)^2}};s_k(c1)^2=\sum_{j=1}^{m}\frac{\left(f_{kj}(c1)-\overline{f}_k(c1)\right)^2}{m};s_k(c2)^2=\sum_{j=1}^{m}\frac{\left(f_{kj}(c2)-\overline{f}_k(c2)\right)^2}{m}$$

It is straightforward to generalize this to the case where there are multiple values of c by using ANOVA. Next, consider the case in which the effect of c on x has the form:

$$x_{ij}(c_j)=h_i(c_j)x_i^0+\gamma_{ij}$$

Where h_i are some functions of c and γ_{ij} is a white noise with the properties as m becomes large:

$$\frac{1}{m}\sum_{j=1}^{m}\gamma_{ij}=0$$
$$\frac{1}{m}\sum_{j=1}^{m}t_{ij'}\gamma_{ij}=0;\forall t$$
$$\frac{1}{m}\sum_{j=1}^{m}\gamma_{ij}\gamma_{i'j}=\gamma^2\delta_{ii'}$$

and:

$$\overline{c}=\frac{1}{m}\sum_{j=1}^{m}c_j$$

Then:

$$\widehat{x}_{ij}(c_j)=x_{ij}(c_j)-\frac{1}{m}\sum_{j=1}^{m}x_{ij}(c_j)=\left[h_i(c_j)-\frac{1}{m}\sum_{j=1}^{m}h_i(c_j)\right]x_i^0+\gamma_{ij}$$

The Pearson Correlation between the value of c at any point in time and x is then:

$$R_i=\frac{\frac{1}{m}\sum_{j=1}^{m}(c_j-\overline{c})\widehat{x}_{ij}(c_j)}{\sqrt{\frac{1}{m}\sum_{j=1}^{m}(c_j-\overline{c})^2}\sqrt{\frac{1}{m}\sum_{j=1}^{m}\widehat{x}_{ij}(c_j)^2}}$$

$$=\frac{\frac{1}{m}\sum_{j=1}^{m}(c_j-\overline{c})\left[\left[h_i(c_j)-\frac{1}{m}\sum_{j=1}^{m}h_i(c_j)\right]x_i^0+\gamma_{ij}\right]}{\sqrt{\frac{1}{m}\sum_{j=1}^{m}(c_j-\overline{c})^2}\sqrt{\frac{1}{m}\sum_{j=1}^{m}\left[\left[h_i(c_j)-\frac{1}{m}\sum_{j=1}^{m}h_i(c_j)\right]^2x_i^{02}+2\gamma_{ij}h(c_j)x_i^0+\gamma_{ij}^2\right]}}=\frac{\frac{1}{m}x_i^0\sum_{j=1}^{m}(c_j-\overline{c})\left[h_i(c_j)-\frac{1}{m}\sum_{j=1}^{m}h_i(c_j)\right]}{\sqrt{\frac{1}{m}\sum_{j=1}^{m}(c_j-\overline{c})^2}\sqrt{\frac{1}{m}\sum_{j=1}^{m}\left[\left[h_i(c_j)-\frac{1}{m}\sum_{j=1}^{m}h_i(c_j)\right]^2x_i^{02}+\gamma_{ij}^2\right]}}$$

$$=\frac{\frac{1}{m}x_i^0\sum_{j=1}^{m}(c_j-\overline{c})\left[h_i(c_j)-\frac{1}{m}\sum_{j=1}^{m}h_i(c_j)\right]}{\sqrt{\frac{1}{m}\sum_{j=1}^{m}(c_j-\overline{c})^2}\sqrt{\gamma^2+\frac{1}{m}\sum_{j=1}^{m}\left[\left[h_i(c_j)-\frac{1}{m}\sum_{j=1}^{m}h_i(c_j)\right]^2x_i^{02}\right]}}$$

In the particular case where the variation of h with c is small or the signal to noise ratio is small so that

$$\gamma^2 >> \frac{1}{m}\sum_{j=1}^{m}\left[\left[h_i\left(c_j\right)-\frac{1}{m}\sum_{j=1}^{m}h_i\left(c_j\right)\right]^2 x_i^{02}\right]$$

Then:

$$R_i \approx \frac{\frac{1}{m}\sum_{j=1}^{m}\left(c_j-\bar{c}\right)\left[h_i\left(c_j\right)-\frac{1}{m}\sum_{j=1}^{m}h_i\left(c_j\right)\right]}{\sqrt{\frac{1}{m}\sum_{j=1}^{m}\left(c_j-\bar{c}\right)^2}\gamma} x_i^0$$

This means that the variation of R over time (i) will be similar to the variation of x if h is independent of i. In the case where the noise is small so that:

$$\gamma^2 << \frac{1}{m}\sum_{j=1}^{m}\left[\left[h_i\left(c_j\right)-\frac{1}{m}\sum_{j=1}^{m}h_i\left(c_j\right)\right]^2 x_i^{02}\right]$$

Then:

$$R_i \approx \frac{\frac{1}{m}\sum_{j=1}^{m}\left(c_j-\bar{c}\right)\left[h_i\left(c_j\right)-\frac{1}{m}\sum_{j=1}^{m}h_i\left(c_j\right)\right]}{\sqrt{\frac{1}{m}\sum_{j=1}^{m}\left(c_j-\bar{c}\right)^2}\sqrt{\frac{1}{m}\sum_{j=1}^{m}\left[\left[h_i\left(c_j\right)-\frac{1}{m}\sum_{j=1}^{m}h_i\left(c_j\right)\right]^2\right]}} \frac{x_i^0}{\left|x_i^0\right|}$$

So that at each point in time the magnitude of R is determined by the dependence of h on c although its sign is determined by the sign of x. in this case, if h is independent of I then the magnitude of R is the same at all time points. In all the cases if h does not depend on c then R will be zero.

Now in the main text, the Spearman correlation coefficient was used instead of the Pearson correlation shown above but the effects may be similar.
